# Tolerance of Protein Folding to a Circular Permutation in a PDZ Domain

**DOI:** 10.1371/journal.pone.0050055

**Published:** 2012-11-21

**Authors:** Greta Hultqvist, Avinash S. Punekar, Angela Morrone, Celestine N. Chi, Åke Engström, Maria Selmer, Stefano Gianni, Per Jemth

**Affiliations:** 1 Department of Medical Biochemistry and Microbiology, Uppsala University, Uppsala, Sweden; 2 Department of Cell and Molecular Biology, Uppsala University, Uppsala, Sweden; 3 Istituto Pasteur -Fondazione Cenci Bolognetti and Istituto di Biologia e Patologia Molecolari del CNR, Dipartimento di Scienze Biochimiche “A. Rossi Fanelli”, Sapienza Università di Roma, Rome, Italy; Russian Academy of Sciences, Institute for Biological Instrumentation, Russian Federation

## Abstract

Circular permutation is a common molecular mechanism for evolution of proteins. However, such re-arrangement of secondary structure connectivity may interfere with the folding mechanism causing accumulation of folding intermediates, which in turn can lead to misfolding. We solved the crystal structure and investigated the folding pathway of a circularly permuted variant of a PDZ domain, SAP97 PDZ2. Our data illustrate how well circular permutation may work as a mechanism for molecular evolution. The circular permutant retains the overall structure and function of the native protein domain. Further, unlike most examples in the literature, this circular permutant displays a folding mechanism that is virtually identical to that of the wild type. This observation contrasts with previous data on the circularly permuted PDZ2 domain from PTP-BL, for which the folding pathway was remarkably affected by the same mutation in sequence connectivity. The different effects of this circular permutation in two homologous proteins show the strong influence of sequence as compared to topology. Circular permutation, when peripheral to the major folding nucleus, may have little effect on folding pathways and could explain why, despite the dramatic change in primary structure, it is frequently tolerated by different protein folds.

## Introduction

Processes such as point mutation, gene duplication and fusion, recombination and circular permutation drive evolution. Circular permutations, where the old termini are sealed and new termini are created at a different site, were considered as rare events since they are difficult to detect by looking only at primary structure. With an increased amount of available 3D structures and structure comparison tools the estimated number of circular permutants has increased drastically. Jung and Lee showed that 14% of the domains in the structural SCOP domain database have at least one circularly permuted “homolog”, i.e., one of these is likely to have arisen through circular permutation [1].

Among the handful of studies on isolated protein domains that have been published, circular permutation often results in more complex kinetic folding mechanisms than for the wild type [2]–[5] and sometimes population of low energy intermediates [6]–[8]. The only exception is the two-state folder chymotrypsin inhibitor 2, where the folding pathway remained the same on circular permutation [9]. In general, more complex folding mechanisms result in accumulation of intermediates and misfolding, which in turn may cause disease and will therefore be disfavoured by evolution [10]. Why then is circular permutation so frequent? Otzen and Fersht suggested that folding of protein domains with diffuse folding nuclei are more likely to be unaffected by circular permutation. Another study showed that if the cleavage site is within the “folding elements”, stretches of amino acids important for early folding events, the protein will not fold, while if located elsewhere it will fold with conserved early folding events [11]. To learn more about how circular permutation affects folding pathways, we analyzed a protein domain with a relatively complex folding pathway, namely the second Postsynaptic density protein-95/Discs large/Zonula Occludens-1 (PDZ) domain from synapse associated protein 97 (SAP97). SAP97 is a member of the membrane-associated guanylate kinase family, and involved in establishing cell polarity [12] and synaptic potentiation [13]. We also compare our results to those from another PDZ domain, PDZ2 from protein tyrosine phosphatase-BL (PTP-BL), an enzyme involved in signal transduction and which carries a number of recognition domains in addition to its catalytic domain [14]. PDZ domains are usually part of such multi domain proteins and have important roles in molecular recognition.

**Figure 1:**
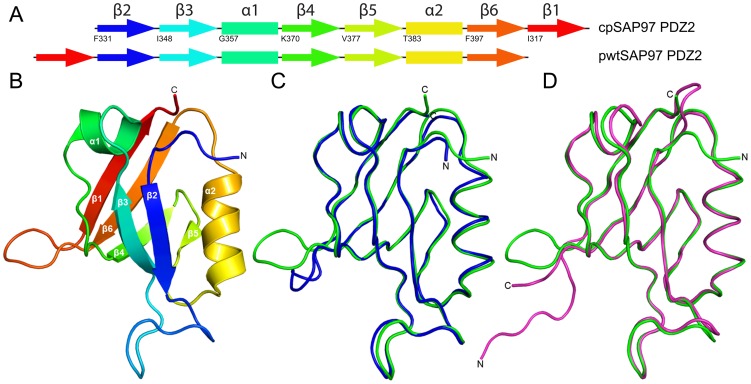
Structure of the circularly permuted SAP97 PDZ2 (cpSAP97 PDZ2). **A.** Schematic picture of the rearrangement of secondary structural elements in cpSAP97 PDZ2. The secondary structure arrangement is naturally occurring in a PDZ domain in green alga [17], [18] and even though it seems modest, had a significant effect on the folding of PTP-BL PDZ2 [6], [7]. **B.** Ribbon representation of the cpSAP97 PDZ2 structure showing the new N and C termini. **C.** Superposition of the two cpSAP97 PDZ2 molecules in the crystal structure, A (green) and B (blue), shown as C_α_ trace. **D.** Superposition of cpSAP97 PDZ2 (green) and pwtSAP97 PDZ2 (pink) shown as C_α_ trace.

PDZ domains are well-characterized globular protein domains of around 90 amino acids with a conserved fold but with substantially different primary structure [15], [16]. In the case of SAP97 PDZ2 and PTP-BL PDZ2 the identity is only 43% but their 3D structures superimposable. PDZ domains consist of six β-strands and two α- helices ordered in the following way: β1- β2- β3- α1- β4- β5- α2- β6 (Figure 1A). There is also a naturally occurring circularly permuted variant of the canonical PDZ domain, where β-strand 1 is placed after β-strand 6 [17], [18]. In the case of PDZ2 from PTP-BL, this circular permutation was engineered and resulted in accumulation of a low-energy intermediate in the folding reaction [6], [7]. Indeed, this permutation stabilized the β-sheet formed by strands β1 and β6 in a region where the early nucleus is formed in the folding reaction of PTP-BL PDZ2 [19], [20].

Wild type PTP-BL PDZ2 is known to fold without any low energy intermediates. On the other hand, folding of SAP97 PDZ2 involves a low energy intermediate, which can be either on- or off-pathway [21], [22]. Therefore, this protein domain offers a good experimental system to probe the effect of circular permutation on a complex folding energy landscape. We have therefore determined the crystal structure and studied the folding pathway of the β6-β1 circular permutant of SAP97 PDZ2 (Figure 1). In contrast to PTP-BL PDZ2, we found that the folding mechanisms for the canonical and circularly permuted SAP97 PDZ2 are remarkably similar.

## Results

### Design of a Circularly Permuted Protein

A circularly permuted (cp) SAP97 PDZ2 domain was generated by fusing the N- and C termini of a pseudo wild-type (pwt) SAP97 PDZ2 [23] with a glycine and serine linker (GSG) between E315 and P405. The new N- and C- termini, K327 and P326, respectively, were selected since they correspond to the terminals of the naturally occurring circular permutant of a PDZ domain from a green alga [17], [18] (see Figure 1A). The same permutation, had a severe effect on the folding of PTP-BL PDZ2 [6], [7].

### The Circularly Permuted and Canonical SAP97 PDZ2 Share the Same Fold

We solved the crystal structure of the cpSAP97 PDZ2 to ensure that the overall structure was not altered by the permutation. The cpSAP97 PDZ2 protein crystallized in the space group C2 with two molecules in the asymmetric unit. The structure was solved by molecular replacement and refined to a resolution of 2.3 Å. In the deposited pdb entry (4AMH), residues Lys13 - Pro91 and Glu95 - Pro106 correspond to the residues Lys327 - Pro405 and Glu315 to Pro326, respectively, in wild-type SAP97-PDZ2. Below, we will refer to the residue numbering of the wild-type protein. In both molecules in the asymmetric unit the residues from Lys327 to Pro 405 via the linker Gly-Ser-Gly and from the next residue Glu315 to Lys324 are ordered. The N-terminal 12 residues including the histidine tag, the thrombin cleavage site and the C-terminal residues (Pro326 in molecule A and Gly325-Pro326 in molecule B) are disordered. The data collection and refinement statistics are shown in Table 1. The cpSAP97 PDZ2 protein structure has the typical PDZ domain fold with six β-strands (β1 to β6) and two α-helices (α1 and α2) (Figure 1B). Superposition of molecules A and B (Figure 1C) shows that the overall root mean square deviation (r.m.s.d) between the A and B molecules is 1.34 Å over 91 C_α_ atoms (Lys327-Pro405-Gly-Ser-Gly-Glu315-Lys324). Minor conformational changes are observed in α2 and the preceding loop as well as in the β2-β3 loop. A larger conformational change is observed in the engineered β6-β1 loop with a maximum deviation of 6.5 Å between the C_α_ atoms of the second glycine in the linker region of the two molecules. The weak electron density and high B-factors for the Gly-Ser-Gly linker and the neighbouring residues in both molecules suggest that this loop is highly flexible. Examination of the crystal contacts shows that the β2-β3 loop and β6-β1 loop in both molecules are involved in crystal packing interactions. Superimposing the cpSAP97 PDZ2 (molecule A) onto the pwtSAP97 PDZ2 (pdb 2X7Z) shows that the structures are very similar (Figure 1D), except for the different N and C-termini, the break in the β1-β2 loop and the new loop connecting β6 to β1. The overall r.m.s.d for 91 aligned C_α_ atoms (Lys327-Lys324) is 0.88 Å. Interestingly, even after moving β1 from the N-terminus to the C-terminus, the orientations of the side-chains in the Ile317-Ile323 region of the cpSAP97 PDZ2 structure are similar to those in the pwtSAP97 PDZ2 structure. As previously observed in the pwtSAP97 PDZ2 structure, Lys324 in the cpSAP97 PDZ2 does not form a salt bridge with Asp396 but forms a hydrogen bond interaction with the side-chain of Thr394.

**Table 1:** Data collection and refinement statistics.

Data collection statistics	
Wavelength (Å)	0.8726
Space group	C2
Cell dimensions	
a, b, c (Å)	106.9, 43.6, 51.7
α, β, γ (°)	90, 90, 103.7
Resolution (Å)^1^	41.3–2.3 (2.35–2.30)
R_merge_ (%)	8.5 (64.7)
I/σ(I)	13.1 (2.2)
Completeness (%)	99.5 (98.9)
Redundancy	3.71 (3.74)
**Refinement statistics**	
Unique reflections (test set)	10415 (521)
R_work_/R_free_ (%)	21.9/26.8
Molecules/asymmetric unit	2
Number of atoms	
Protein	1386
Water	53
Other	14
Average B factors	
Protein	40.9
Water	36.2
Other	44.8
RMSD from ideal bond length (Å)	0.008
RMSD from ideal bond angle (°)	1.264
Ramachandran plot statistics	
Residues in favoured regions (%)	94.5
Residues in additional allowed regions (%)	5.5
Residues in disallowed regions (%)	0.0

1 Values in parentheses represent the highest resolution bin.

**Figure 2:**
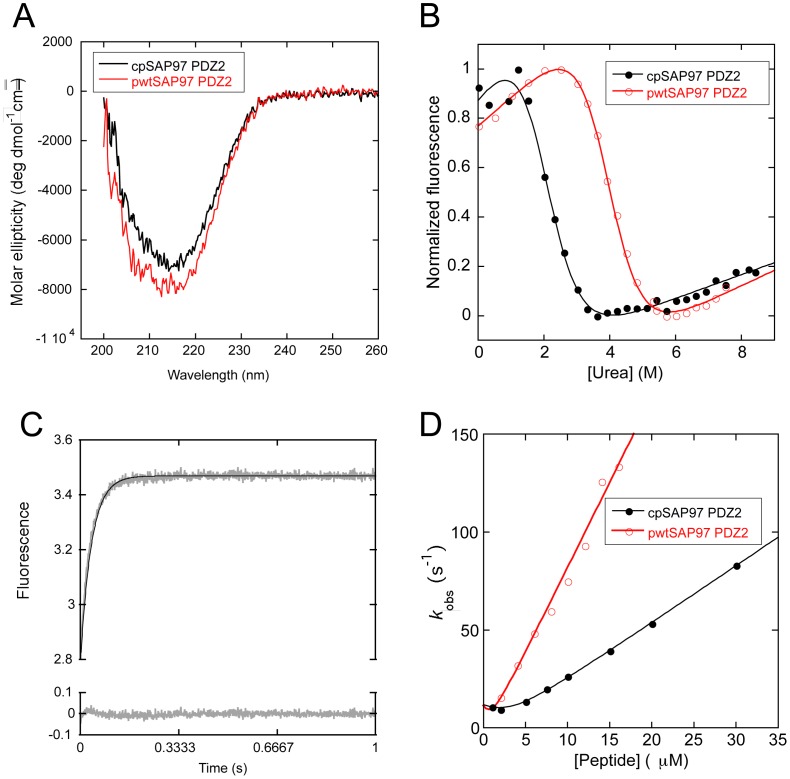
Equilibrium and peptide binding data for cp- and pwtSAP97PDZ2. **A.** Far-UV circular dichroism spectra of the cp- and pwtSAP97PDZ2. **B.** Urea denaturation curve of cp- and pwtSAP97 PDZ2, respectively. The *m*_D-N_ -value (1.2 kcal mol^−1^M^−1^) was shared in the curve fitting to illustrate the similarities between the curves. See Table 2 for fitted parameters. **C.** cpSAP97 PDZ2 binding trace at 10 µM peptide fitted to a single exponential function. Residuals are shown below the trace. **D.** Observed rate constants for the binding of the peptide LQRRRETQV to cp- and pwtSAP97 PDZ2 in 50 mM potassium phosphate, pH 7.5, plotted against peptide concentration. Fitting was done with the general equation for a second order bimolecular association [50]. The association rate constant, *k*_on_ (slope of the linear region of the curve), decreased from 8.7±0.3 to 2.9±0.02 µM^−1^s^−1^ upon circular permutation, while the dissociation rate constant remained basically the same.

Circular dichroism experiments confirmed that the circularly permuted protein is folded and contains similar ratios of secondary structural elements as the pwtSAP97 PDZ2 in our experimental conditions (Figure 2A).

**Table 2:** Equilibrium parameters for the stability of cpSAP97 PDZ2 and kinetic parameters for its association with the peptide LQRRRETQV.

	pwtSAP97PDZ2	cpSAP97PDZ2
*m*_D-N_ –value (kcal mol^−1^ M^−1^)	1.20±0.08^1^	1.20±0.08^1^
*m*_D-N_ –value (kcal mol^−1^ M^−1^)	1.04±0.04^2^	1.4±0.2^2^
[Urea]_50%_(M)	3.93±0.06^1^	2.00±0.09^1^
[Urea]_50%_(M)	3.94±0.03^2^	2.1±0.1^2^
Δ*G*_D-N_ (kcal/mol)	4.7±0.3^1^	2.4±0.2^1^
*k*_on_ (µM^−1^s^−1^)	8.7±0.3^3^	2.93±0.02
*k*_off_ (extrapolated) (s^−1^)	2.4±2.3	3.0±0.2
*k*_off_ (displacement) (s^−1^)	2.1±0.1	1.8±0.2

1 Shared *m*_D-N_ –value in the curve fitting.

2 Free fitting.

3 From ref. [51].

### The Circular Permutation Reduces the Stability of the Protein

To compare the thermodynamic stability of the pwt- and cpSAP97 PDZ2 we did equilibrium denaturation experiments in 50 mM potassium phosphate pH 7.5 by varying the urea concentration and measuring the fluorescence of the single tryptophan (Trp342) present in the respective protein (Figure 2B). The equilibrium constants were obtained by fitting the data to the general equation for solvent denaturation of a protein according to a two-state mechanism [24], since the denaturation curves displayed a simple sigmoidal transition with no evidence for intermediate states populated at equilibrium. Since the two proteins have very similar sequence and overall fold, the *m*_D-N_ - value, which reflects the change in solvent accessible surface area on denaturation, can be shared in the fitting process. The data show that the circular permutation has decreased the stability of the protein from 4.7±0.03 to 2.4±0.09 kcal/mol (Table 2).

### The cpSAP97 PDZ2 Retains Binding to the Wild Type Ligand

From kinetic binding experiments, SAP97 PDZ2 is known to bind to a peptide, LQRRRETQV, generated from the C-terminus of the human papillomavirus 18 (HPV-18) E6 protein [23]. We showed that cpSAP97 PDZ2 retains its binding to this peptide (Figure 2C and D) by measuring the fluorescence change from Trp342 upon binding, using the stopped flow technique. The association rate constant, *k*_on_, was one-third of that of the pwt SAP97 PDZ2, whereas the dissociation rate constant, *k*_off_, remained essentially unchanged, as determined from a separate displacement experiment [23] (Table 2).

### Observed Folding Rate Constants are Consistent with a Multi-step Mechanism

**Figure 3:**
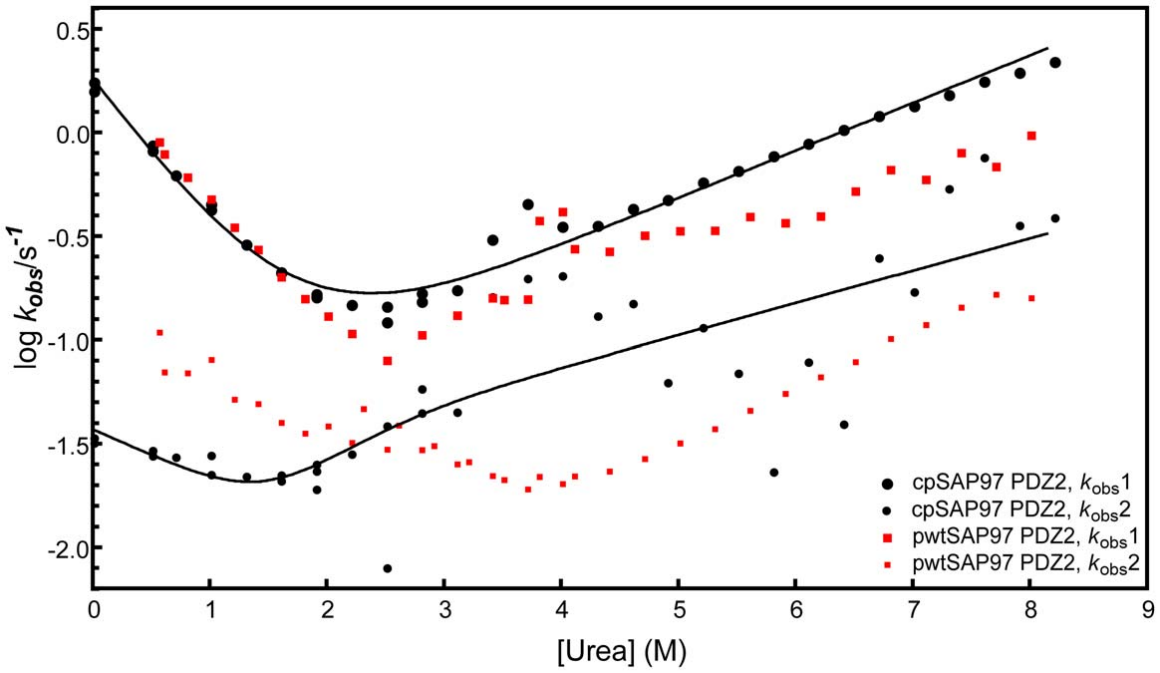
Analysis of the two different phases in kinetic folding experiments. Chevron plots of cp- and pwtSAP97 PDZ2 in 50 mM potassium phosphate, pH 7.5, showing the rate constants corresponding to the two observed phases. The black continuous line shows an on-pathway fit to the kobs values for cpSAP97 PDZ2. The fits to off-pathway and triangular schemes were equally good and are not shown. For cpSAP97 PDZ2 the phase with the largest amplitude is always the fastest one, while for pwtSAP97 PDZ2 the phase with the largest amplitude is the fastest one between urea concentrations 0–2.5 M and then it switches to be the slower one, hence, what is referred to as the main phase in Figure 5 is represented by both kobs1 and kobs2.

Having demonstrated that the structure and function of the cpSAP97 PDZ2 were intact, we investigated its folding pathway. Folding kinetics can be studied in urea-induced unfolding experiments, and buffer-induced refolding experiments in the stopped flow fluorimeter, using the same tryptophan probe (Trp342) as in the binding experiments. Kinetic traces were biphasic, both for the refolding and unfolding reactions, resulting in two observed rate constants (*k*_obs_). The observed refolding phases were independent of protein concentration at low urea concentration within a range of 0.2–5 µM, thus excluding protein aggregation events. By plotting the *k*_obs_ value against the urea concentration on a semi- logarithmic scale a chevron plot is generated. In Figure 3, the chevron plot from the experiments in 50 mM potassium phosphate, pH 7.5 at various urea concentrations is shown, both for pwt- and cpSAP97 PDZ2. When double exponential (un)folding kinetics is observed, the folding mechanism is more complex than a two state reaction. However, to distinguish among different reaction schemes is very difficult. The three simplest reaction schemes that result in double exponential folding kinetics are (i) a two-step folding with an on-pathway intermediate, (ii) a two-step folding with an off-pathway intermediate, and (iii) a triangular scheme with an on-pathway intermediate as well as a direct formation of the native state from the denatured state [25]–[28]. Haq et al. [18] suggested that the data for pwtSAP97 PDZ2 were best fitted to a two-step folding scenario with an off-pathway intermediate at 25°C, or an on-pathway or triangular scheme at 37°C [21]. The (un)folding rate constants for cpSAP97 PDZ2 can be nicely fitted to all of the three above suggested reaction schemes (on-pathway fit shown in Figure 3). Therefore, this data set was not sufficient to distinguish between different potential folding pathways for the cpSAP97 PDZ2.

### The cpSAP97 PDZ2 Folds with Two Compact and Two Denatured Like Species

**Figure 4:**
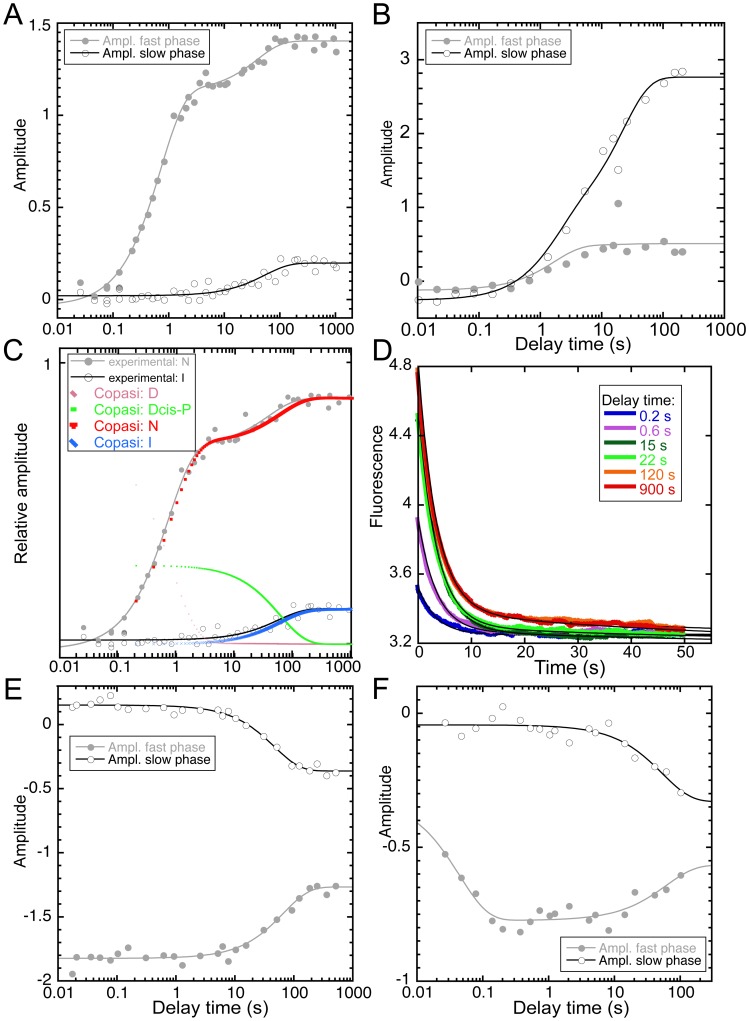
Double jump experiments of cpSAP97 PDZ2 and pwtSAP97 PDZ2. **A.** Plot of the amplitudes for the two observed rate constants in an interrupted refolding experiment for cpSAP97 PDZ2. See panel D for examples of raw data. **B.** Plot of the amplitudes for the two observed rate constants in an interrupted refolding experiment for pwtSAP97 PDZ2. The data is from a previous publication [21]. **C.** The experimental data from Figure 4A together with simulated traces for the square model. The simulation was done using Copasi [29] and using the rate constants in Table S2. The initial distribution of the D states, 72% D and 28% D_cis-P_, was calculated from the ratio of D and D_cis-P_ at equilibrium in the interrupted unfolding experiment. The excellent fit illustrates that the square model can explain our experimental data. **D.** Examples of experimental traces from interrupted refolding of cpSAP97 PDZ2 after various delay times. The traces were fitted to a double exponential curve (black) with shared rate constants and kinetic amplitudes plotted versus delay time (panel A). **E.** Plot of the amplitudes for the two observed rate constants in an interrupted unfolding experiment for cpSAP97 PDZ2. **F.** Plot of the amplitudes for the two observed rate constants in an interrupted unfolding experiment for pwtSAP97 PDZ2. The delay time plotted on the x-axis is the incubation time of the first mix. For example, in an interrupted refolding experiment it is the time the protein is allowed to refold before unfolding is initiated by the second mixing event.

**Figure 5:**
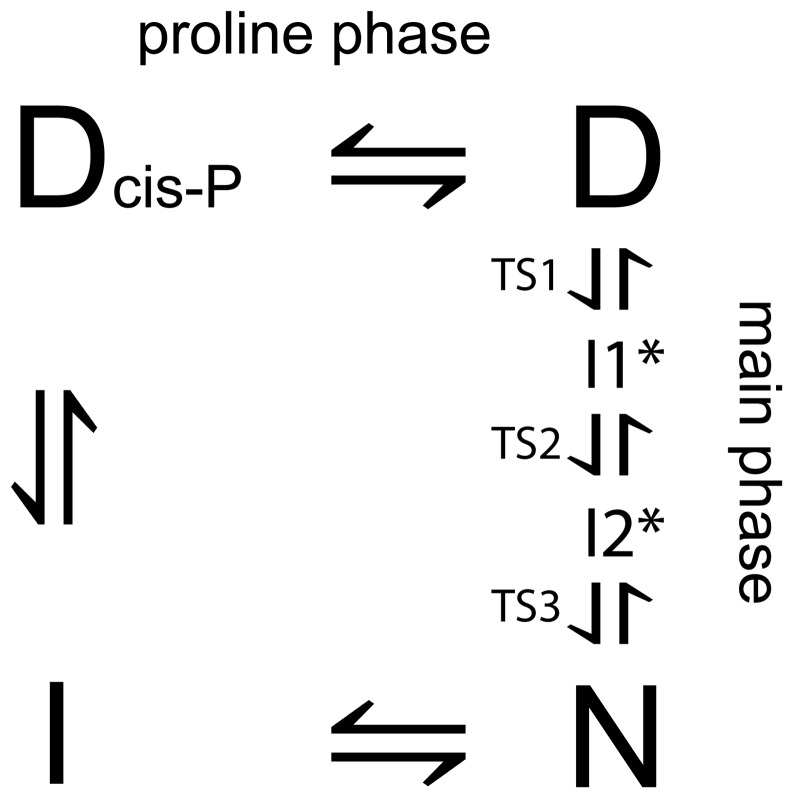
Unifying folding reaction scheme for pwt- and cpSAP97 PDZ2. We have illustrated that the folding of cpSAP97 PDZ2 and its natural canonical version pwtSAP97 PDZ2 can be described by this unifying folding scheme with four states, D_cis-P_, D, I and N. D_cis-P_ and D are denatured states, N is the native state, whereas I is a compact state with native-like burial of hydrophobic residues. The high energy intermediares I_1_* and I_2_* seem to be a conserved feature among most PDZ domains [22], [44] and give rise to the observed non-linearities for the observed (un)folding rate constants for the main phase.

More information on the folding pathway of cpSAP97 PDZ2 was obtained by performing interrupted refolding and interrupted unfolding experiments. In these experiments refolding or unfolding is interrupted after various delay times and then the protein is unfolded/refolded again. This powerful technique allows detection of populations of individual species with time after mixing. We observed two kinetic phases at long delay times in the interrupted refolding experiment (thus, at equilibrium) showing that there are two distinct states at equilibrium (Figures 4A and 4D), which are denoted I and N in the scheme in Figure 5. The state that unfolds faster (I) is formed in a clear double exponential fashion. Similarly, the interrupted unfolding experiments revealed two distinct states at high urea concentration (Figure 4E), denoted D and D_cis-P_ in Fig. 5. Here, the rapidly refolding species unfolds in a clearly double exponential way, but the rise from 0 to maximum amplitude is faster than the dead-time of the stopped flow instrument in the sequential mix setup (the minimum delay time between the first and the second mix being in the order of 10 ms). Together, these experiments illustrate that at least four states are involved in the folding of cpSAP97 PDZ2. The simplest reaction scheme to describe such folding data is a square model with two more compact states (I and N) and two denatured, expanded species (D and D_cis-P_). Our suggested folding model for cpSAP97 PDZ2 is shown in Figure 5. In the interrupted refolding experiment the fast phase would be represented by the transition from the denatured state D to the native state N (illustrated by the first phase in Figure 4A) but also by the transition between D and D_cis-P_. Because of the low rate constants, as discussed below, we postulate this heterogeneity in denatured states to arise from a denatured state with at least one proline in cis conformation (hence D_cis-P_). The slow phase in Fig. 4A would then represent the transition from D_cis-P_ to the equilibrium intermediate I. In Figure 4C, we demonstrate that our data on cpSAP97 PDZ2 can be fitted to the square model by using the program Copasi [29], which simulates how the concentrations of the different species change with time in the folding reaction. Normal curve fitting was difficult to employ since the equation describing the square model is very complex.

### Proline Isomerization is the Likely Cause of the Slow Phase

The folding of some proteins containing prolines is slowed down due to the proline cis-trans isomerization, which gives rise to an additional folding phase [30], [31]. Some of these proteins have been reported to fold according to a square scheme [32]. The cpSAP97 PDZ2 has three prolines that are located at positions 326, 343 and 405. Hence, it is possible that one of the phases in our suggested square model comes from a proline phase, as outlined below. From the interrupted unfolding experiments we found that the fractions of D and D_cis-P_ at 4 M urea, 12.5 mM HCl, 2.5 mM potassium phosphate, were 78% and 22%, respectively. These numbers were used when fitting data to the interrupted un/refolding experiments with Copasi (Figure 4C). The observed ratio is similar to those previously reported for prolines in cis and trans position in small peptides and other proteins [33], [34]. Furthermore, from our interrupted refolding experiment, the rate of interconversion between D and D_cis-P_ was also similar to that previously reported for proline isomerization [35]. These results together with the proposed square-folding scheme for the cpSAP97 PDZ2 suggest that the proline cis-trans isomerization is the likely cause for the slow kinetic phase. While addition of Pro cis-trans isomerases has been employed to confirm Pro phases, results from these experiments may sometimes be inconclusive due to, for example, the specificity of the enzyme and isomerization of non-Pro peptide bonds [36]. Given the complexity of the observed kinetics for cpSAP97 PDZ2, we chose not to perform such experiments.

### The Canonical pwtSAP97 PDZ2 also Folds According to a Square Reaction Scheme

Results from the interrupted refolding experiments for pwtSAP97 PDZ2 [21] (replotted in Figure 4B) and cpSAP97 PDZ2 initially appear to be different due to the lack of obvious transition for pwtSAP97 PDZ2 that corresponds to the main folding phase. However, a possible explanation for this are the similar rates between D_cis-P_ to D and D to N, respectively. In fact, since we argue that the transition between D and D_cis-P_ is a proline phase, this phase is likely to have the same rate constants independently of protein and conditions. Therefore, since the observed folding rate constant of the main phase in the conditions used in the two interrupted refolding experiments is ten-fold lower for the canonical pwtSAP97 PDZ2, it becomes similar to the rate constants for the proline isomerization. Therefore, we hypothesized that there is a proline phase also in the folding of pwtSAP97 PDZ2 that previously escaped detection, since an interrupted unfolding experiment was not included in the previous analysis [21]. To compare the pwt- and cpSAP97 PDZ2 folding pathways, we therefore did an interrupted unfolding experiment for pwtSAP97 PDZ2. This experiment clearly confirmed that pwtSAP97 PDZ2, similarly to cpSAP97 PDZ2, has two distinct states at high urea (Figure 4F). Thus, the simplest folding scheme for the pwtSAP97 PDZ2 would also be a square model (Figure 5).

### Intermediates are Less Populated in the Circular Permutant Compared to the Wild Type

By comparing the chevron plots of cpSAP97 PDZ2 and pwtSAP97 PDZ2 at the most native like conditions (which is at 0 M urea) (Figure 3) we could see that the folding rate constant of native protein (top phase) is similar for pwt and cpSAP97 PDZ2. On the other hand, the phase leading to the intermediate has a lower folding rate constant for the circular permutant, thereby reducing the formation of intermediate during the folding process of cpSAP97 PDZ2.

### The Kinetics for Direct Formation of Native from Denatured Protein is Conserved between cpSAP97 PDZ2 and Other PDZ Domains

**Figure 6:**
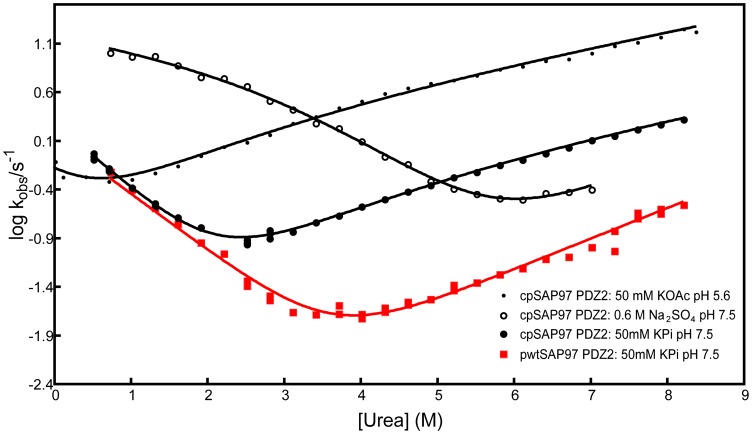
Chevron plots of the main phases of cp- and pwtSAP97 PDZ2 under different conditions. The main phase is the *k*_obs_ value with the largest amplitude. Rollovers in the refolding and unfolding arm of the chevron plots can be detected when altering between stabilizing and destabilizing buffers, respectively. These rollovers illustrate switches between the rate limiting transition states of the (un)folding reaction. Fitting was done using *β*_T_–values obtained from a curve fit with 6 different PDZ domains in a previous study [19] and the good fit to the data for the circular permutant illustrates that the positions of the folding transition states along the reaction coordinate is similar for all PDZ domains, including the circular permutant. See Table S1 for the best fit parameters. The 0.6 M Na_2_SO_4_ buffer also contained 50 mM potassium phosphate, pH 7.5, while the 50 mM potassium acetate buffer, pH 5.6, contained KCl to keep the ionic strength at the same value for all experiments.

In the kinetic folding experiments of cpSAP97 PDZ2 the main phase (with the highest amplitude) is the phase reflecting direct formation of native protein. The observed rate constant for this phase shows a refolding rollover if measured in stabilizing conditions (0.6 M sodium sulfate) and an unfolding rollover when analysed in destabilizing conditions (50 mM sodium acetate, pH 5.6) (Figure 6). Such non-linearities in chevron plots are indications of changes in rate limiting transition states for (un)folding [28]. The degree of solvent accessible surface for theses transition states is described by the β_T_ value, obtained by curve fitting to three state models. The native state N has a β_T_ value of 1 and the denatured state D a value of 0. We have previously shown that PDZ domains fold via three conserved transition states, and hence can be fitted with three shared β_T_- values [22]. Our data for the cpSAP97 PDZ2 fitted well to the β_T_- values (0.17, 0.65 and 0.86, respectively) found in Hultqvist et.al [19], suggesting that the formation of native cpSAP97 PDZ2 follows a similar path as pwtSAP97 PDZ2. The data from the curve fitting can be found in Table S1.

The chevron plots for pwt- and cpSAP97- PDZ2 measured under identical conditions are similar, but with a general shift to lower stability of the main phase due to increased unfolding rate constants for the circularly permuted protein (Figures 3 and 6). Φ- values of 0 or 1 are not associated with the same caveats as intermediate values [37]. The change in *k*_u_ and identical *k*_f_, therefore suggest a Φ- value for circular permutation close to 0, or in structural terms, that the site of circular permutation has not formed native contacts in the transition state for folding of pwtSAP97 PDZ2.

## Discussion

Folding pathways of circularly permuted proteins have been studied in a limited number of cases [2], [4]–[9], [38], [39] and in only one of these has the folding pathway remained the same as for the native protein [9]. It has been argued that changes in folding pathway due to circular permutation depend on the folding nucleus; a diffuse folding nucleus covering most of the protein is less likely to change the folding pathway compared to a regional compact nucleus [9], [40]. In agreement with this notion, Gianni and co-workers demonstrated that circular permutation of PTP-BL PDZ2 resulted in stabilization of an intermediate [7].

The folding mechanism of PTP-BL PDZ2 has been thoroughly investigated by Φ-value analysis and constrained molecular dynamics simulations [19], to estimate the extent of formation of native contacts in the transition state for folding. PTP-BL PDZ2 folds with an early rather compact regional nucleus and a late, very native like transition state [19], [20]. Its early folding nucleus consists of β-strands 1, 4 and 6. For PTP-BL PDZ2, the same circular permutation was made as the one in the present study (i.e., based on the naturally occurring circularly permuted PDZ domain D1pPDZ [17]), but with a different outcome. Thus, by linking β1 and β6 in PTP-BL PDZ2, this early nucleus is stabilized, which is reflected in a higher folding rate constant but also significant stabilization of an intermediate, which is likely to be off-pathway [6]. It is believed that such intermediates are dis-favoured by natural selection because of the increased risk for misfolding [10].

It was recently suggested that the relation between the position of the cleavage site and active site in circular permutants is important for whether the folding pathways change due to the permutation [41]. The site of our permutation is one amino acid away from the GLGF site, which is conserved among all PDZ domains and involved in binding of the backbone and C-terminus of the protein ligand [42]. However, while our data do not directly address the effect of permutation in the binding site, we note that SAP97 PDZ2 is not affected by the circular permutation but its homolog PTP BL PDZ2 displays a dramatic change in kinetic folding mechanism.

For SAP97 PDZ2, circular permutation increased the unfolding rate constant but the folding rate constant (D to N transition, Figure 5) remained unchanged. Effectively, this corresponds to a Φ-value of zero, both at the site of linkage (new turn between β1 and β6) and at the sites of the new N and C-termini (loop between β1 and β2). In other words, these structural elements have not started to form native contacts in the rate limiting transition state for folding. Furthermore, the rate constant for formation of the intermediate (D_cis-P_ to I in Figure 5) was decreased upon circular permutation resulting in a lower maximum concentration of intermediate during the folding reaction. Thus, the result of the circular permutation is very different for the structurally very similar domains, PTP-BL PDZ2 and SAP97 PDZ2, and the basis for the difference is found in their early folding events.

To sum up, our results show how a circular permutation neither alters the structure (Figure 1) nor significantly affects the function (Figure 2) of the protein, SAP97 PDZ2. We further demonstrate that the canonical protein and the circular permutant fold via a similar mechanism (Figure 5), and that the rate of formation of the low energy intermediate has decreased in the circular permutant. These data illustrate the general feasibility of circular permutation as a mechanism for molecular evolution and, as suggested earlier [9], show that such events are most likely to be successful in regions of the protein that are not part of a folding nucleus.

## Materials and Methods

### Cloning, Expression and Purification

#### Cloning

The cDNA for the circular permutant of human SAP97 PDZ2, residues 327–405 connected to residues 315–326 via a GSG linker (see Figure 1A), was ordered from Geneart. Two additional mutations as compared to wild type SAP97 PDZ2 were present in the circularly permuted construct: I342W, as a probe for fluorescence, and C378A, to avoid formation of disulfide bridges. Both mutations have been shown to only have minimal effects on the wild type SAP97 PDZ2 [23]. The cDNA construct was cloned into the EcoRI/BamHI sites of a modified pRSET vector (Invitrogen), which added an N- terminal MHHHHHLVPRGS tag to the expressed protein. This His tag has previously been shown not to affect the stability nor binding of PDZ domains [23], [43], [44]. The expressed product is hereafter referred to as cpSAP97 PDZ2. The canonical variant, pwtSAP97 PDZ2, refers to amino acids 311–407 of the same protein and with the same mutations (I342W, C378A) as used in previous studies [21], [23].

#### Expression

The vector was transformed into *Escherichia coli* BL21-DE3 pLyS cells that grew on LB- agar plates under selection of ampicillin (100 µg/ml) and chloramphenicol (35 µg/ml) at 37°C overnight. From the plates colonies where transferred to liquid LB culture at 37°C under selection of 50 µg/ml ampicillin. At an A_600_ of ∼0.6, protein expression was induced with 1 mM isopropyl-β-D-1-thiogalactopyranoside (IPTG) and grown for 3 more hours before harvesting by centrifugation.

#### Purification for kinetic experiments

The cell pellet was resuspended and frozen in 50 mM potassium phosphate, pH 7.0. After thawing, the cells were disrupted by ultrasonication followed by centrifugation (35 000 g) for 1 hour. The resulting supernatant was filtered through a 0.2 µm filter and incubated with Ni-NTA agarose, Qiagen, for 30 minutes. The agarose was then washed with 50 mM potassium phosphate, 25 mM imidazole, pH 7.5, until the A_280_ was close to 0. The protein was eluted with 50 mM potassium phosphate, 250 mM imidazole at pH 7.5. The eluate was dialysed against 50 mM potassium phosphate pH 7.0 and loaded onto an S-column (GE healthcare), and eluted with a 0–500 mM NaCl gradient. The resulting protein sample was concentrated and dialysed against 50 mM potassium phosphate, pH 7.5. The mass and purity of the protein were analysed through matrix- assisted laser desorption ionization time-of-flight mass spectrometry and SDS-PAGE, respectively.

#### Purification for crystallization

The cell pellet was thawed and resuspended in 50 mM Tris-HCl, pH 7.5, 150 mM NaCl, 0.1% Triton X-100, 5 mg lysozyme, containing complete protease inhibitor (Roche, Germany). The cells were lysed by sonication and the lysate was clarified by centrifugation (23 500 g) for 45 min at 4°C. The supernatant was filtered and loaded onto a Bio-Rad Econo-Pac gravity flow column containing Ni-Sepharose™ High Performance (GE Healthcare, Sweden) pre-equilibrated with 50 mM Tris-HCl, pH 7.5, 150 mM NaCl, 10 mM imidazole and incubated for 1 h at 4°C. The column was washed with 50 mM Tris-HCl, pH 7.5, 150 mM NaCl, 40 mM imidazole and the His-tagged cpSAP97 PDZ2 was eluted with 50 mM Tris-HCl, pH 7.5, 150 mM NaCl, 250 mM imidazole. The fractions containing His-tagged cpSAP97 PDZ2 protein were pooled and concentrated to a final volume of 5.0 ml using a Vivaspin 5 kDa cut-off concentrator (Sartorius Stedim Biotech, Germany). The protein was further purified through a HiLoad™ 16/60 Superdex™ 75 prep grade column (GE Healthcare, Sweden) using gel filtration buffer (50 mM Tris-HCl, pH 7.5, 100 mM NaCl). The peak fractions containing cpSAP97 PDZ2 protein were pooled and concentrated to 20 mg/ml using a Vivaspin 5 kDa cut-off concentrator (Sartorius Stedim Biotech, Germany).

### Structure Determination

#### Crystallization

A single crystal of cpSAP97 PDZ2 protein grew after several weeks at 4°C by vapour diffusion in a sitting drop composed of 1.5 µl protein (20 mg/ml His-tagged cpSAP97 PDZ2 in gel filtration buffer) and 1.5 µl reservoir solution (0.1 M MES, pH 6.0, 2.4 M ammonium sulfate) (Grid Screen Ammonium Sulfate, Hampton Research). For data collection, the crystal was cryoprotected by soaking in the reservoir solution supplemented with 20% glycerol for 1 min and flash frozen in liquid nitrogen.

#### Data collection and processing

X-ray diffraction data were collected on beam line ID23-2 at ESRF, Grenoble, France. Data were processed in space group C2 using XDS [45]. Initial phases were obtained by molecular replacement with the program Phaser [46] using the pwtSAP97 PDZ2 structure, pdb 2X7Z [21] as a search model. The final complete model obtained by iterative rounds of model building using Coot [47] and refinement using PHENIX [48] has R_work_ of 21.9% and R_free_ of 26.8%. The quality of the structure was assessed using MolProbity [49]. Refinement statistics can be found in Table 1. The refined coordinates have been deposited in the pdb with accession number 4AMH. Structure Figures were prepared using PyMOL (The PyMOL Molecular Graphics System, Version 1.2r1, Schrödinger, LLC).

#### Circular dichroism

Far-UV circular dichroism was measured using a Jasco J-810 spectropolarimeter for wavelengths between 200 and 260 nm, with 20 µM protein in 50 mM potassium phosphate, pH 7.5.

### Stability Experiments

Urea induced equilibrium denaturation experiments were carried out with 5 µM protein in 50 mM potassium phosphate pH 7.5 at 25°C and varying urea concentrations. After excitation at 280 nm, the emission between 300 and 400 nm from Trp342, Tyr349 and Tyr399 was monitored with an SLM 4800 spectrofluorimeter (SLM Aminco, Urbana, IL). The resulting curve from plotting fluorescence at 350 nm against the urea concentration was fitted to the standard equation for solvent denaturation [24].

### Binding Experiments

Ligand-binding kinetic experiments were carried out with 1 or 3 µM protein in 50 mM potassium phosphate, pH 7.5 at 10°C and at various peptide (LQRRRETQV) concentrations. All kinetic experiments were carried out on an SX-20MV stopped flow instrument (Applied Photophysics, Leatherhead, UK). After excitation (280 nm) of Trp342 the change in emission above 320 nm upon binding was followed using a cut-off filter. The observed rate constants, *k*_obs_, were obtained after fitting the resulting trace to a single exponential equation [23]. These rate constants were plotted against peptide concentration and the data fitted to the general equation for a second-order bimolecular association [50] to get the association rate constant, *k*_on_, and dissociation rate constant, *k*_off_. Experiments with the His-tag cleaved of by thrombin were performed to confirm that the His-tag did not affect the binding.

### Folding Experiments

#### Single-mixing experiments

Kinetic folding and unfolding rate constants were measured in the SX-20MV stopped flow spectrometer by 1∶10 mixing of protein solution and urea-buffer solutions of varying concentrations of urea, at 25°C. The final protein concentrations in these single-jump kinetic experiments were 3 µM when measured in 50 mM potassium phosphate, 1.2 µM when measured in 50 mM potassium acetate, 8.6 mM KCl, pH 5.6 and 0.6 µM when measured in 0.4 M or 0.6 M sulfate, 50 mM potassium phosphate pH 7.5. Individual points with protein concentrations between 0.2 and 10 µM were tested to ensure that the results were not dependent on protein concentration.

For the refolding experiments the protein stock solutions were in 5.5 or 8 M urea and the corresponding final buffer. No refolding traces were measured at pH 5.6. The samples for all folding experiments were excited at 280 nm and the fluorescence emission through a 320 nm cut-off filter was recorded. The measured time courses in all of the refolding and unfolding experiments were fitted to either a single or a double exponential equation to obtain the observed rate constants. The rate constants were plotted on a semi-logarithmic plot against urea concentration, to obtain a chevron plot. The logarithms of the microscopic rate constants were assumed to have linear dependence on the urea concentration [24]. The chevron plot obtained in 50 mM potassium phosphate, pH 7.5 was fitted to equations for a sequential three-state mechanism with an on-pathway or off-pathway intermediate or to a triangular scheme [21]. The main phase of the chevron plots were also analysed individually assuming a sequential pathway with two high-energy intermediates and three transition states, where the switch from TS1 to TS2 causes the refolding arm rollover and the switch from TS2 to TS3 causes the unfolding arm rollover for the cpSAP97 PDZ2. When no change in the rate-limiting step (rollover) occurred for the main phase in the chevron plot, it was fitted to a two state equation with transition state 2 (TS2) as rate limiting When a change in the rate-limiting step could be detected, as a rollover in one of the arms, the chevron curve was fitted to a three state equation. All equations can be found in ref. [22].

#### Interrupted refolding

In interrupted refolding experiments 2.4 µM of protein in 4 M urea, 10 mM HCl (no buffer) was refolded by mixing 1∶1 with 0.8 M Na_2_SO_4_, 100 mM potassium phosphate pH 7.5. After different delay times the protein was unfolded by mixing 1∶1 with 9.2 M urea, 0.4 M Na_2_SO_4_, 50 mM potassium phosphate, pH 7.5 and the resulting kinetic trace was recorded. Thus, the refolding was done in 50 mM potassium phosphate, pH 7.5, 2 M urea, 0.4 M Na_2_SO_4_, and the subsequent unfolding in the same buffer but with a final urea concentration of 6.6 M. The resulting kinetic traces could be fitted to a double exponential equation. Since all the points were measured in the same experimental conditions but just with different delay times, the observed rate constants should be identical. Hence, in one double jump experiment, we fitted all the obtained kinetic traces to shared rate constants to get the amplitudes at different delay times. These amplitudes were plotted against delay time and fitted to a single or double exponential equation.

#### Interrupted unfolding

In interrupted unfolding experiments of cpSAP97 PDZ2, 2.4 µM of protein in 5 mM potassium phosphate, pH 7.5 was unfolded by mixing 1∶1 with 8 M Urea, 25 mM HCl. After different delay times the protein was refolded by mixing 1∶1 with 0.8 M Na_2_SO_4_, 100 mM potassium phosphate, pH 7.5 and the resulting kinetic trace was recorded. For pwtSAP97 PDZ2, 2.4 µM of protein in 2 M urea, 5 mM potassium phosphate, pH 7.5, was unfolded by mixing 1∶1 with 8 M Urea, 25 mM HCl. After different delay times the protein was refolded by mixing 1∶1 with 100 mM potassium phosphate, pH 7.5 and the resulting kinetic trace was recorded. The resulting traces were analysed as previously described for the interrupted refolding experiments.

## Supporting Information

Table S1 Best fit folding parameters to chevron plots of the main phase of cpSAP97 PDZ2 and pwtSAP97 PDZ2 under different conditions. Fitting was done using the β_T_–values obtained in a previous study (ref. [22] in the paper), where six PDZ domains were found to fold via a unifying mechanism. See Fig. 6 for experimental data and fitted curves.
(DOCX)

Table S2 Rate constants used in the Copasi simulation in Figure 4C of experimental data (Figure 4A) to the square model.
(DOCX)

## References

[1] Jung J, Lee B (2001) Circularly permuted proteins in the protein structure database. Prot. Sci. 10: 1881–1886 doi:10.1110/ps.05801 10.1110/ps.05801 PMC2253204 11514678

[2] Viguera AR, Serrano L, Wilmanns M (1996) Different folding transition states may result in the same native structure. Nat. Struct. Mol. Biol. 3: 874–880 doi:10.1038/nsb1096-874 10.1038/nsb1096-874 8836105

[3] Li L, Shakhnovich EI (2001) Different circular permutations produced different folding nuclei in proteins: a computational study. J. Mol. Biol. 306: 121–132 doi:10.1006/jmbi.2000.4375 10.1006/jmbi.2000.4375 11178898

[4] Lindberg M, Tångrot J, Oliveberg M (2002) Complete change of the protein folding transition state upon circular permutation. Nat. Struct. Mol. Biol. 9: 818–822 doi:10.1038/nsb847 10.1038/nsb847 12368899

[5] Cellitti J, Llinas M, Echols N, Shank EA, Gillespie B, et al (2007) Exploring subdomain cooperativity in T4 lysozyme I: Structural and energetic studies of a circular permutant and protein fragment. Prot. Sci. 16: 842–851 doi:10.1110/ps.062628607 10.1110/ps.062628607 PMC2206633 17400926

[6] Ivarsson Y, Travaglini-Allocatelli C, Morea V, Brunori M, Gianni S (2008) The folding pathway of an engineered circularly permuted PDZ domain. Protein Eng. Des. Sel. 21: 155–160 doi:10.1093/protein/gzm077 10.1093/protein/gzm077 18175779

[7] Ivarsson Y, Travaglini-Allocatelli C, Brunori M, Gianni S (2009) Engineered Symmetric Connectivity of Secondary Structure Elements Highlights Malleability of Protein Folding Pathways. J. Am. Chem. Soc. 131: 11727–11733 doi:10.1021/ja900438b 10.1021/ja900438b 19722594

[8] Gianni S, Ivarsson Y, Simone AD, Travaglini-Allocatelli C, Brunori M, et al (2010) Structural characterization of a misfolded intermediate populated during the folding process of a PDZ domain. Nat. Struct. Mol. Biol. 17: 1431–1437 doi:10.1038/nsmb.1956 10.1038/nsmb.1956 21076399

[9] Otzen DE, Fersht AR (1998) Folding of Circular and Permuted Chymotrypsin Inhibitor 2: Retention of the Folding Nucleus. Biochemistry 37: 8139–8146 doi:10.1021/bi980250g 9609709 10.1021/bi980250g

[10] Chiti F, Dobson CM (2009) Amyloid formation by globular proteins under native conditions. Nat. Chem. Biol. 5: 15–22 doi:10.1038/nchembio.131 10.1038/nchembio.131 19088715

[11] Arai M, Maki K, Takahashi H, Iwakura M (2003) Testing the relationship between foldability and the early folding events of dihydrofolate reductase from Escherichia coli. J. Mol. Biol. 328: 273–288. 10.1016/s0022-2836(03)00212-2 12684013

[12] Nakagawa T, Futai K, Lashuel HA, Lo I, Okamoto K, et al (2004) Quaternary structure, protein dynamics, and synaptic function of SAP97 controlled by L27 domain interactions. Neuron 44: 453–467 doi:10.1016/j.neuron.2004.10.012 15504326 10.1016/j.neuron.2004.10.012

[13] Funke L, Dakoji S, Bredt DS (2005) Membrane-associated guanylate kinases regulate adhesion and plasticity at cell junctions. Annu. Rev. Biochem. 74: 219–245 doi:10.1146/annurev.biochem.74.082803.133339 10.1146/annurev.biochem.74.082803.133339 15952887

[14] Saras J, Engström U, Góñez LJ, Heldin CH (1997) Characterization of the interactions between PDZ domains of the protein-tyrosine phosphatase PTPL1 and the carboxyl-terminal tail of Fas. J. Biol. Chem. 272: 20979–20981. 10.1074/jbc.272.34.20979 9261095

[15] Nourry C, Grant SGN, Borg J-P (2003) PDZ Domain Proteins: Plug and Play! Sci. STKE 2003: re7 doi:10.1126/stke.2003.179.re7 12709532 10.1126/stke.2003.179.re7

[16] Jemth P, Gianni S (2007) PDZ domains: Folding and binding. Biochemistry 46: 8701–8708 doi:10.1021/bi7008618 17620015 10.1021/bi7008618

[17] Liao D-I, Qian J, Chisholm DA, Jordan DB, Diner BA (2000) Crystal structures of the photosystem II D1 C-terminal processing protease. Nat. Struct. Mol. Biol. 7: 749–753 doi:10.1038/78973 10.1038/78973 10966643

[18] Ivarsson Y, Travaglini-Allocatelli C, Brunori M, Gianni S (2008) Folding and Misfolding in a Naturally Occurring Circularly Permuted PDZ Domain. J. Biol. Chem. 283: 8954–8960 doi:10.1074/jbc.M707424200 10.1074/jbc.M707424200 18263589

[19] Gianni S, Geierhaas CD, Calosci N, Jemth P, Vuister GW, et al (2007) A PDZ domain recapitulates a unifying mechanism for protein folding. Proc. Natl. Acad. Sci. U.S.A 104: 128–133 doi:10.1073/pnas.0602770104 10.1073/pnas.0602770104 PMC1765422 17179214

[20] Calosci N, Chi CN, Richter B, Camilloni C, Engström Å, et al (2008) Comparison of successive transition states for folding reveals alternative early folding pathways of two homologous proteins. Proc. Natl. Acad. Sci. U.S.A 105: 19241–19246 doi:10.1073/pnas.0804774105 10.1073/pnas.0804774105 PMC2614746 19033470

[21] Haq SR, Jürgens MC, Chi CN, Koh C-S, Elfström L, et al (2010) The plastic energy landscape of protein folding: a triangular folding mechanism with an equilibrium intermediate for a small protein domain. J. Biol. Chem. 285: 18051–18059 doi:10.1074/jbc.M110.110833 10.1074/jbc.M110.110833 PMC2878566 20356847

[22] Hultqvist G, Pedersen SW, Chi CN, Strømgaard K, Gianni S, et al (2012) An expanded view of the protein folding landscape of PDZ domains. Biochem. Biophys. Res. Commun. 421: 550–553 doi:10.1016/j.bbrc.2012.04.042 10.1016/j.bbrc.2012.04.042 22521641

[23] Chi CN, Bach A, Engström Å, Wang HQ, Strømgaard K, et al (2009) A Sequential Binding Mechanism in a PDZ Domain. Biochemistry 48: 7089–7097 doi:10.1021/bi900559k 19496620 10.1021/bi900559k

[24] Fersht A (1999) Structure and mechanism in protein science: a guide to enzyme catalysis and protein folding. W.H. Freeman.

[25] Parker MJ, Spencer J, Clarke AR (1995) An integrated kinetic analysis of intermediates and transition states in protein folding reactions. J. Mol. Biol. 253: 771–786 doi:10.1006/jmbi.1995.0590 10.1006/jmbi.1995.0590 7473751

[26] Wildegger G, Kiefhaber T (1997) Three-state model for lysozyme folding: triangular folding mechanism with an energetically trapped intermediate. J. Mol. Biol. 270: 294–304 doi:10.1006/jmbi.1997.1030 10.1006/jmbi.1997.1030 9236130

[27] Capaldi AP, Shastry MCR, Kleanthous C, Roder H, Radford SE (2001) Ultrarapid mixing experiments reveal that Im7 folds via an on-pathway intermediate. Nat. Struct. Mol. Biol. 8: 68–72 doi:10.1038/83074 10.1038/83074 11135674

[28] Gianni S, Ivarsson Y, Jemth P, Brunori M, Travaglini-Allocatelli C (2007) Identification and characterization of protein folding intermediates. Biophys. Chem. 128: 105–113 doi:10.1016/j.bpc.2007.04.008 10.1016/j.bpc.2007.04.008 17498862

[29] Hoops S, Sahle S, Gauges R, Lee C, Pahle J, et al (2006) COPASI–a COmplex PAthway SImulator. Bioinformatics 22: 3067–3074 doi:10.1093/bioinformatics/btl485 17032683 10.1093/bioinformatics/btl485

[30] Kiefhaber T, Quaas R, Hahn U, Schmid FX (1990) Folding of ribonuclease T1. 1. Existence of multiple unfolded states created by proline isomerization. Biochemistry 29: 3053–3061. 2110823 10.1021/bi00464a023

[31] Jackson SE, Fersht AR (1991) Folding of chymotrypsin inhibitor 2. 2. Influence of proline isomerization on the folding kinetics and thermodynamic characterization of the transition state of folding. Biochemistry 30: 10436–10443. 1931968 10.1021/bi00107a011

[32] Salahuddin A (1984) Proline peptide isomerization and protein folding. J. Biosci. 6: 349–355 doi:10.1007/BF02703893

[33] Stellwagen E (1979) Proline peptide isomerization and the reactivation of denatured enzymes. J. Mol. Biol. 135: 217–229 doi:10.1016/0022-2836(79)90348-6 10.1016/0022-2836(79)90348-6 529287

[34] Larive CK, Rabenstein DL (1993) Dynamics of cis/trans isomerization of the cysteine6-proline peptide bonds of oxytocin and arginine-vasopressin in aqueous and methanol solutions. J. Am. Chem. Soc. 115: 2833–2836 doi:10.1021/ja00060a033

[35] Kiefhaber T, Schmid FX (1992) Kinetic coupling between protein folding and prolyl isomerization. II. Folding of ribonuclease A and ribonuclease T1. J. Mol. Biol. 224: 231–240. 10.1016/0022-2836(92)90586-9 1548701

[36] Pappenberger G, Aygün H, Engels JW, Reimer U, Fischer G, et al (2001) Nonprolyl cis peptide bonds in unfolded proteins cause complex folding kinetics. Nat. Struct. Biol. 8: 452–458 doi:10.1038/87624 10.1038/87624 11323723

[37] Fersht AR, Sato S (2004) Φ-Value analysis and the nature of protein-folding transition states. Proc Natl Acad Sci U S A 101: 7976–7981 doi:10.1073/pnas.0402684101 15150406 10.1073/pnas.0402684101 PMC419542

[38] Bulaj G, Koehn RE, Goldenberg DP (2004) Alteration of the disulfide-coupled folding pathway of BPTI by circular permutation. Prot. Sci. 13: 1182–1196 doi:10.1110/ps.03563704 10.1110/ps.03563704 PMC2286756 15096625

[39] Haglund E, Lindberg MO, Oliveberg M (2008) Changes of protein folding pathways by circular permutation. Overlapping nuclei promote global cooperativity. J. Biol. Chem. 283: 27904–27915 doi:10.1074/jbc.M801776200 10.1074/jbc.M801776200 18562318

[40] Clementi C, Jennings PA, Onuchic JN (2001) Prediction of folding mechanism for circular-permuted proteins. J. Mol. Biol. 311: 879–890 doi:10.1006/jmbi.2001.4871 10.1006/jmbi.2001.4871 11518537

[41] Capraro DT, Gosavi S, Roy M, Onuchic JN, Jennings PA (2012) Folding Circular Permutants of IL-1β: Route Selection Driven by Functional Frustration. PLoS ONE 7: e38512 doi:10.1371/journal.pone.0038512 22693643 10.1371/journal.pone.0038512 PMC3367917

[42] Chi CN, Bach A, Strømgaard K, Gianni S, Jemth P (2012) Ligand binding by PDZ domains. BioFactors (Oxford, England). Available: http://www.ncbi.nlm.nih.gov/pubmed/22674855. Accessed 7 Sep 2012. 10.1002/biof.1031 22674855

[43] Gianni S, Engström Å, Larsson M, Calosci N, Malatesta F, et al (2005) The kinetics of PDZ domain-ligand interactions and implications for the binding mechanism. J. Biol. Chem 280: 34805–34812 doi:10.1074/jbc.M506017200 10.1074/jbc.M506017200 16049001

[44] Chi CN, Gianni S, Calosci N, Travaglini-Allocatelli C, Engström Å, et al (2007) A conserved folding mechanism for PDZ domains. FEBS Lett. 581: 1109–1113 doi:10.1016/j.febslet.2007.02.011 10.1016/j.febslet.2007.02.011 17316619

[45] Kabsch W (2010) XDS. Acta Crystallogr. D Biol. Crystallogr. 66: 125–132 doi:10.1107/S0907444909047337 10.1107/S0907444909047337 PMC2815665 20124692

[46] McCoy AJ, Grosse-Kunstleve RW, Adams PD, Winn MD, Storoni LC, et al (2007) Phaser crystallographic software. J. Appl. Crystallogr. 40: 658–674 doi:10.1107/S0021889807021206 10.1107/S0021889807021206 PMC2483472 19461840

[47] Emsley P, Lohkamp B, Scott WG, Cowtan K (2010) Features and development of Coot. Acta Crystallogr. D Biol. Crystallogr. 66: 486–501 doi:10.1107/S0907444910007493 10.1107/S0907444910007493 PMC2852313 20383002

[48] Adams PD, Afonine PV, Bunkóczi G, Chen VB, Davis IW, et al (2010) PHENIX: a comprehensive Python-based system for macromolecular structure solution. Acta Crystallogr. D Biol. Crystallogr. 66: 213–221 doi:10.1107/S0907444909052925 10.1107/S0907444909052925 PMC2815670 20124702

[49] Chen VB, Arendall WB, Headd JJ, Keedy DA, Immormino RM, et al (2009) MolProbity: all-atom structure validation for macromolecular crystallography. Acta Crystallogr. D Biol. Crystallogr. 66: 12–21 doi:10.1107/S0907444909042073 10.1107/S0907444909042073 PMC2803126 20057044

[50] Malatesta F (2005) The study of bimolecular reactions under non-pseudo-first order conditions. Biophys. Chem. 116: 251–256 doi:10.1016/j.bpc.2005.04.006 10.1016/j.bpc.2005.04.006 15896898

[51] Haq SR, Chi CN, Bach A, Dogan J, Engström Å, et al (2011) Side-Chain Interactions Form Late and Cooperatively in the Binding Reaction between Disordered Peptides and PDZ Domains. J. Am. Chem. Soc. 134: 599–605 doi:10.1021/ja209341w 10.1021/ja209341w 22129097

