# Supplementary material for: Tolerance of Protein Folding to a Circular Permutation in a PDZ Domain
Source: PLoS One. 2012 Nov 21;7(11):e50055. doi: 10.1371/journal.pone.0050055 (PMC3503759; doi:10.1371/journal.pone.0050055)
Supplement: Table S1 — Best fit folding parameters to chevron plots of the main phase of cpSAP97 PDZ2 and pwtSAP97 PDZ2 under different conditions. Fitting was done using the βT–values obtained in a previous study (ref. [22] in the paper), where six PDZ domains were found to fold via a unifying mechanism. See Fig. 6 for experimental data and fitted curves. (DOCX) [file pone.0050055.s001.docx]

**Supporting Table S1. Best fit folding parameters to chevron plots of the main phase of cpSAP97 PDZ2 and pwtSAP97 PDZ2 under different conditions.** Fitting was done using the β_T_–values obtained in a previous study (ref. [19] in the paper), where six PDZ domains were found to fold via a unifying mechanism. See Fig. 6 for experimental data and fitted curves.

|  | cpSAP97 PDZ2: 50 mM KOAc pH 5.6 | cpSAP97 PDZ2: 0.6 M Na_2_SO_4_ pH 7.5 | cpSAP97 PDZ2: 50 mM KP_i_ pH 7.5 | pwtSAP97 PDZ2: 50 mM KP_i_ pH 7.5 |
| --- | --- | --- | --- | --- |
| *k*_F1_ (s^-1^) | - | 15.9±0.8 | - | - |
| *k*_F2_ (s^-1^) | 0.44±0.05 | 280±80 | 1.93±0.08 | 1.4±0.1 |
| *k*_U1_ (s^-1^) | - | 0.0032±0.0006 | - | - |
| *k*_U2_ (s^-1^) | 0.30±0.03 | - | 0.015±0.0006 | 0.00077±0.00008 |
| *k*_U3_ (s^-1^) | 1.0±0.4 | - | 0.15±0.01 | - |
| *m*_D-N_ (kcal mol^-1^ M^-1^) | 1.5±0.2 | 1.18±0.06 | 1.44±0.03 | 1.38±0.03 |
