# Supplementary material for: Tolerance of Protein Folding to a Circular Permutation in a PDZ Domain
Source: PLoS One. 2012 Nov 21;7(11):e50055. doi: 10.1371/journal.pone.0050055 (PMC3503759; doi:10.1371/journal.pone.0050055)
Supplement: Table S2 — Rate constants used in the Copasi simulation in Figure 4C of experimental data (Figure 4A) to the square model. (DOCX) [file pone.0050055.s002.docx]

**Supporting Table S2. Rate constants applied in the simulation of experimental data (Figure 4A) to the theoretical square model by Copasi in Figure S1.**

|  | ***k*_forw_** (s^-1^) | ***k*_rev_** (s^-1^) |
| --- | --- | --- |
| **D - N** | 1.2 | 0.001 |
| **N’- N** | 0.001 | 0.00014 |
| **D_cis-P_ – N’** | 0.007 | 0.000016 |
| **D_cis-P_ - D** | 0.01 | 0.0039 |
